# Mapping African Swine Fever and Highly Pathogenic Avian Influenza Outbreaks along the Demilitarized Zone in the Korean Peninsula

**DOI:** 10.1155/2024/8824971

**Published:** 2024-05-30

**Authors:** Changmin Im, Andrew Curtis, Daesub Song, Oh Seok Kim

**Affiliations:** ^1^ Department of Geography Graduate School of Korea University SeoulRepublic of Korea; ^2^ GIS Health and Hazards Lab Department of Population and Quantitative Health Sciences School of Medicine Case Western Reserve University Cleveland OH 44106USA; ^3^ College of Veterinary Medicine and Research Institute for Veterinary Science Seoul National University SeoulRepublic of Korea; ^4^ Department of Geography Education College of Education Korea University SeoulRepublic of Korea; ^5^ Institute of Future Land Korea University SeoulRepublic of Korea

## Abstract

The Korean Demilitarized Zone (DMZ) is one of the world's most preserved habitats for wild animals and migratory birds. The area also plays a major role in the spread of infectious animal diseases, in particular, African swine fever (ASF) and highly pathogenic avian influenza (HPAI). These outbreaks threaten the livelihood of local livestock farms, not infrequently. In this paper, we explore these relatively under-researched diseases by modeling and mapping ASF and HPAI risks in tandem using MaxEnt, a machine-learning algorithm. The results show robust predictive power with high area under the curve values, of 0.92 and 0.99, respectively. We found that precipitation from spring to early summer and solar radiation in winter were essential in explaining the potential distribution of ASF, but land use contributed little. Thus, understanding only wild boars' habitat preferences may not be sufficient in preventing ASF epidemics. HPAI risks were shaped by precipitation and mean temperature from winter to spring and land use. Areas with high ASF and HPAI risks were primarily found in forest and agricultural lands, respectively. The DMZ included many high-risk areas, indicating that the DMZ could lead to a broader regional spread of ASF and HPAI in the peninsula. Thus, our results highlight the essential role of cross-border collaboration and the combination of environmental and epidemiological insights in strategies to control ASF and HPAI risks within and surrounding the DMZ.

## 1. Introduction

The accurate prediction of disease spread is vital in understanding potential risk and where to target intervention strategies [1]. Modeling is always dependent on accurate and timely data release; a sophisticated disease surveillance system can be used to develop near real-time geographic insights in terms of where a disease is emerging and where and how quickly it is spreading [2, 3, 4]. Low-data quality results in poor model accuracy, reducing their effective use in operational planning [5]. While the reasons for poor data quality are many, for example, not having the resources to collect data in a timely and accurate manner, political boundaries provide one of the more challenging obstacles [6, 7]. In areas where diseases spread across borders of traditionally conflicting powers, often the only way to predict how they will spread is to use computer models that can extrapolate outcomes from limited input data [8, 9]. In this paper, we use one such modeling strategy to predict where disease spread might happen in one of the world's most vulnerable areas, the border transition areas between South and North Korea. The inner border area contains the Demilitarized Zone (hereafter DMZ) established by the Armistice Agreement in 1953 [10].

Somewhat ironically, the strong military presence and the restriction of activities have also resulted in one of the world's most undisturbed habitats for flora and fauna; a total of 5,929 species of animals and plants have been found here, of which 101 are considered endangered [11]. This natural preserve, which provides an excellent habitat for a diverse array of animals and migratory birds, also plays a role in the spread of infectious animal diseases, especially African swine fever (hereafter ASF) and highly pathogenic avian influenza (hereafter HPAI) [12, 13, 14] which are of global concern due to their potential for severe economic and ecological impacts [15, 16, 17].

In recent years, both diseases have occurred annually throughout the peninsula [18, 19]. Thanks to surveillance systems, we can detect and respond to diseases early enough to limit impacts. In September 2019, the government reported for the first time an ASF outbreak to the World Organisation for Animal Health (WOAH); the case resulted in the culling of approximately 0.4 million pigs [20]. The initial ASF outbreak in Korea began near the DMZ and has steadily advanced southward. The implementation of the wide-area fence in the DMZ serves as a distinct sign of ASF's southward trajectory, emphasizing the gravity of the situation. HPAI has frequently occurred since 2003, especially in the winter of 2016–2017 when there was a cull of approximately 40 million poultry due to the outbreak [21], leading to substantial losses on poultry farms [22]. As a result of these previous outbreaks, there is a need for detailed spatial outbreak prediction modeling, though the lack of North Korean data has been a limitation [13, 23]. As a result, though there have been different spatial epidemiological studies that have analyzed ASF and HPAI outbreaks in South Korea [22, 24, 25, 26], a few have included areas of North Korea. In the past, risk maps of ASF and HPAI have identified possible infectious disease transmission risks based on statistical probability [27, 28, 29, 30]. MaxEnt provides an alternative approach that overcomes some of these data deficiencies as it employs a machine-learning algorithm and has strength in modeling ecological niche and disease risk with presence-only data based on underlying environmental characteristics, such as land use and local climate [31, 32, 33, 34].

In this paper, we will use MaxEnt and the latest government data along the greater DMZ region to produce risk maps for ASF and HPAI with the aim of identifying potential transmission routes between South Korea and North Korea.

More specifically, we aim to answer the following questions:

1. Which environmental variables are more important in explaining ASF and HPAI risks?
2. What are the characteristics of areas with high risk of ASF and HPAI?
3. In terms of epidemiology, what are the implications of having the DMZ in the peninsula?

It is important to note that the present study is about wild animals and does not consider farm animals. ASF is considered endemic in this case study as it has only to do with wild animals; ASF is not endemic regarding farm animals in South Korea. HPAI is not endemic either in the wild or on farms at the national level, even though it occurs regularly (i.e., sporadic outbreaks).

## 2. Materials and Methods

**Figure 1:**
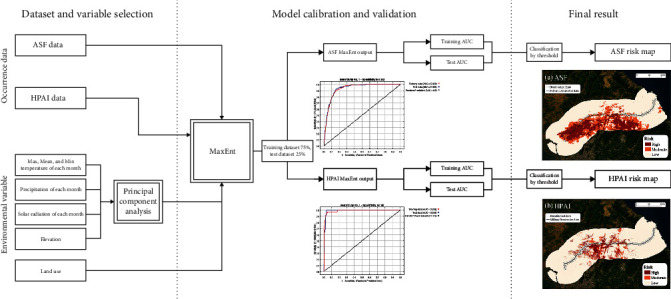
Schematic diagram of methodology.

The overall framework of this study includes (1) data collection and processing; (2) model fitting and evaluation; and (3) generation of the ASF and HPAI risk maps and their overlay, as illustrated in Figure 1. Each step's specific details are presented in the subsequent sections.

### 2.1. Study Area

**Figure 2:**
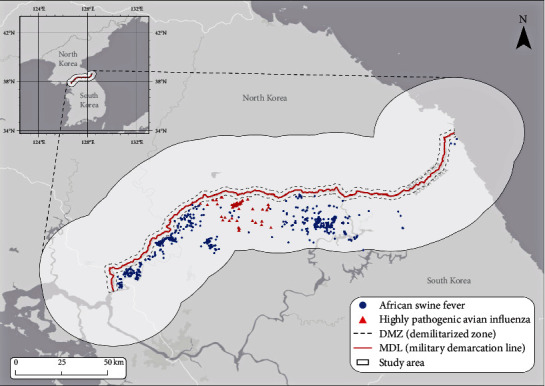
Study area and distribution of ASF (669) and HPAI (144) positive cases.

The study area is the 30 km buffer zone from the military demarcation line, covering both parts of South Korea and North Korea. The 30 km buffer distance allows for including all ASF and HPAI data points (Figure 2). Therefore, our study area includes the DMZ, the zone that since the Korean War has been inaccessible due to the ongoing military tension between the South and the North. As a result, the DMZ is largely underdeveloped, resulting in many abandoned agricultural lands (some of which have been recovered as artificial wetlands) and unmanaged forests (where some have become old-growth if not burned by wildfire) [10, 35, 36, 37]. Municipalities adjacent to the DMZ are also underdeveloped because the South Korean government has legally limited any large-scale regional development in the neighborhood [36]. As a result, the nonmilitary human involvement in the study area is largely agriculture (mostly rice paddies) and livestock farming facilities. This political (and military) rationale for land use has also produced ideal ecological niches for some viruses.

The South Korean side of the study area includes two administrative provinces, namely Gyeonggi (west) and Gangwon (east). Gyeonggi is the most populous province (13.4 million people) in South Korea [38]; Gangwon is mainly characterized by forests and then agricultural lands [39]. The North Korean side covers three provinces: South Hwanghae (west), North Hwanghae (center), and Kangwon (east). The large plains in the former two provinces have resulted in agricultural land use [40]. South Hwanghae is the largest producer of rice, maize, and soybean in North Korea [41]. Kangwon province is mostly mountainous similar to the adjacent Gangwon province of South Korea.

### 2.2. Input Data

#### 2.2.1. ASF and HPAI Cases

Geocoded data of ASF and HPAI carcasses were obtained from the central and local governments, respectively [42, 43], and only the positive cases were used in this study. The data are only available for limited time periods: there were 669 ASF cases from October 2019 to July 2020 (approximately 10 months), while 144 HPAI cases were reported between January 2021 and March 2021 (approximately 2 months). Their spatial distribution is illustrated in Figure 2.

As for ASF, soldiers (who have access to the DMZ and other restricted military areas) and local residents/hunters (who have considerable local knowledge and were hired by local governments and the Ministry of Environment) patrolled the area to locate carcasses. Once identified, locations were documented as coordinates and cadastral-level addresses using GPS, followed by biosecurity measures to prevent further spreading. Dates are twofold: one for the identification of the carcass at the site and the other for the test result. Additional information, such as sex, terrain, age, etc., was also documented when available. Finally, these were directly reported to the government.

As for HPAI, local farmers usually reported to the local government when dead birds were found in their or their neighbors' rice paddy fields. Then, government officials visited the site to photograph the carcasses and document the coordinates and cadastral-level addresses, before sending the specimens to the National Wildlife Disease Control Center for HPAI positive/negative testing and species identification.

ASF has continued to spread since October 2019, and it is uncertain when this diffusion will stop [44]. This ongoing spread is another reason why analyzing the geographic pattern of ASF is vital. ASF being endemic in South Korea inherently means it is also endemic in our study area, i.e., the inner-border region. This justifies our MaxEnt application because it meets the equilibrium requirement. Our input ASF data (October 2019–August 2020) have obvious limitations because the ongoing epidemic by definition means these data are not complete.

HPAI data (January–March 2021), on the other hand, are not fully endemic and thus violating the equilibrium assumption of MaxEnt. HPAI epidemics are seasonal because they are mainly driven by migratory birds, namely *Asner albifrons* (greater white-fronted goose), *Cygnus cygnus* (Whooper swan), *Grus japonensis* (red-crowned crane), and *Antigone vipio* (white-naped crane). The first HPAI case was reported on 30 December 2020 near the western inner border (Gyeonggi Daily 2020), and the spread ended on 30 March 2021 (Asia Business Daily 2021). HPAI spread can be portrayed as a series of localized outbreaks, i.e., “semi-endemic.” Although limited, our data offer the best spatial resolution for HPAI in the study area. Thus, we argue that MaxEnt is applicable to this case study due to its strength in dealing with spatially biased data and showing considerable resilience in maintaining predictive accuracy. However, the results must be interpreted cautiously.

#### 2.2.2. Environmental Variables

We employed climatic, elevation, and land use data when modeling MaxEnt for ASF and HPAI (Table 1). Climatic data (1 by 1 km) were obtained from the World Climate Database (WorldClim) [45]. Elevation and land-use maps (30 by 30 m) were provided by the Ministry of Environment of South Korea. The land use included four categories: urban, agriculture, forest, and others. The WorldClim data were resampled to 30 m spatial resolution to match the resolution of elevation and land-use maps. The 30 m spatial resolution is the most appropriate for modeling because the ASF and HPAI data points were closely located to each other. Further, to remove multicollinearity, we conducted a principal components analysis (PCA) on all the variables, except for land use due to it being categorical. We conducted PCA on the environmental layers for the study area and then extracted principal component (PC) values for each occurrence location. Meaningful PCs were selected as input data based on eigenvalue-one criterion, scree test and cumulative variance [46, 47].

Climatic data were selected and combined differently for each disease. In South Korea, four seasons are typically defined by average temperature thresholds: (a) spring, above 5°C; (b) summer, above 20°C; (c) fall, below 20°C; and (d) winter, below 5°C [48]. As ASF spreads throughout all seasons in South Korea, we have used the entire monthly WorldClim data for the ASF to run PCA. As for HPAI, we have used the WorldClim data of December–April to cover our HPAI data (January–March 2021) to portray winter and spring. The additional months, December and April, were added to test a possible lag effect between climate and the disease. Studies suggested that the climatic conditions before and after the main disease period can have a preparatory or consequential impact on disease transmission [49, 50, 51]. Only the mean temperature was used in the research and did not include maximum or minimum temperature based on the existing study [52]. The final PCs are used as input data for MaxEnt modeling to explain the spread of ASF and HPAI, respectively.

### 2.3. Modeling ASF and HPAI using MaxEnt

MaxEnt identifies the distribution with the maximum entropy to predict where a virus is likely to be present [30, 53, 54]. To calculate this distribution, a maximum likelihood approach was applied followed by the sequential-update algorithm which began with a uniform distribution. One or more weights of predictor variables were sequentially modified to maximize the average log probability of the presence samples [55]. Its predictive accuracy had previously been proven to be high in similar research [56].

To evaluate MaxEnt application's performance (not goodness-of-fit), we used 75% of the ASF and HPAI cases to train the model, while the rest was independently used for testing the predictive outcome; all case points were combined with 10,000 randomly selected background points. A receiver operating characteristic (ROC) and the associated area under the ROC curve (AUC) indicate an overall goodness-of-fit of the MaxEnt model. AUC values often used in assessing the accuracy of infectious disease predictions range from 0 to 1 where 0.5 indicates random prediction and higher values mean more accurate results [55, 57, 58].

We also assessed the contribution each input data had on explaining ASF and HPAI. MaxEnt assesses variables' contributions in two ways: permutation importance and percent contribution. The former is more suitable for assessing the contribution because it is determined based on the final model by permuting values of each variable among all training points, whereas the latter only considers a single iteration [59]; therefore, we assessed the contribution of PCs and land use based on permutation importance only. In other words, final PCs of each disease might be fewer than PCs inputted for MaxEnt modeling because a PC is likely to have little permutation importance in MaxEnt even though it meets the eigenvalue-one, scree test, and cumulative variance criteria in PCA.

MaxEnt provides two thresholds to determine the presence of viruses [60]. The first threshold is designed to maximize the accuracy of the model based on the given presence data [61]. The second threshold, on the other hand, takes into account both the given presence data and omitted observations in the study area [32, 62]. As a result, the latter leads to a more balanced and moderate outcome compared to the former approach. Thus, we used the former to delineate “high” risk areas, while the latter to delineate “moderate” and “low” risk areas. When there is an overlap between high and moderate areas, it is classified as high. Finally, we overlaid the high-risk maps of ASF and HPAI to identify areas exposed to both viruses.

## 3. Results

### 3.1. Model Performance

All AUCs were greater than 0.91, suggesting that the MaxEnt results were reliable in being able to predict potential ASF and HPAI risks in the greater DMZ region. The HPAI model showed higher AUCs than the ASF model, though this does not necessarily mean that the former is better than the latter. AUCs of training and those of testing showed little difference for both ASF and HPAI (Table 2).

### 3.2. Variable Contributions

The relative contributions (i.e., permutation importance) of the input data to the MaxEnt modeling of ASF and HPAI are summarized in Table 2, where PCs and land cover were used as the input data. After PCA, the first four PCs (from the first PC to the fourth PC, hereafter PC1–4) and the first three PCs (from the first PC to the third PC, hereafter PC1–3) were selected for ASF and HPAI, respectively (Figures S1 and S2; Tables S1 and S2). To fully understand the impact of the environmental variables, it is necessary to refer to the PCA results, including factor loadings (Tables S1, S2, S3, and S4).

1. ASF.

All PC1–4 contributed to explaining the ASF spread, but land use was not included in the final model due to its minor contribution during the initial run. PC3 (31.97%) contributed the most, followed by PC4 (26.90%), PC1 (22.87%), and PC2 (18.26%) (Table 2). PC3 meant precipitation from spring to early summer and solar radiation in winter. PC4 indicated mean temperature from late spring to early summer and solar radiation in winter. PC1 indicated mean temperature and solar radiation from early spring through late autumn, strongly related to elevation. Lastly, PC2 showed precipitation from summer to winter and mean temperature in winter (Table S3). In short, in explaining the ASF risks in the study area, the most important environmental variables are precipitation, mean temperature, and solar radiation.

(2) HPAI.

PC2 (48.09%) contributed the most in explaining the HPAI spread, followed by PC1 (37.88%) and land use (14.02%); PC3 was not included in the final model due to its minor contribution (Table 2). Noting that the research only considers winter and spring seasons for HPAI, PC2 indicated precipitation; PC1 represented mean temperature associated with elevation (Table S4). In summary, the most important environmental variables in explaining HPAI risks were precipitation, temperature, and land use. Lastly, we identified the lag effect in December but not April.

### 3.3. Geographical Distribution of ASF and HPAI

**Figure 3:**
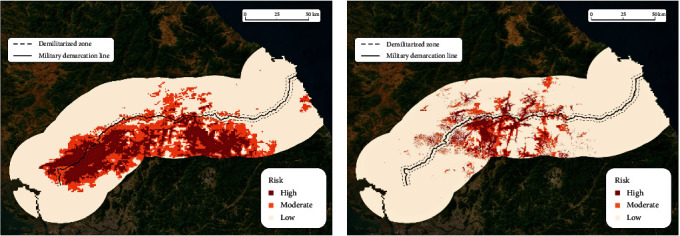
MaxEnt maps of (a) ASF and (b) HPAI.

The two empirically driven thresholds (15.52% and 17.97%) categorized the ASF risks into high, moderate, and low areas. High-risk areas in the South were concentrated in the western and central regions (Figure 3(a)), and most of them (75.05%) belong to forestlands (Table 3). Despite employing climatic and land-use data of North Korea, ASF risks were found to be much lower in the North than in the South, while those risk areas in the North were primarily concentrated in the western region along the inner border (Figure 3(a); Table 4).

Similarly, the other set of two thresholds (4.73% and 6.19%) classified HPAI risks into high, moderate, and low. High-risk areas in the South were concentrated in the central region (Figure 3(b)), and most of them (56.64%) belong to agriculture (Table 3). In terms of area, HPAI had fewer high-risk areas than ASF (Figure 3(b); Table 4). HPAI's high-risk areas in North Korea were worth 33.1% of South Korea, whereas ASF's high-risk areas in North Korea were worth 14.8% of South Korea. The DMZ included twice as many high-risk areas as North Korea (Table 4).

## 4. Discussion

The highly inaccessible DMZ and its underdeveloped neighboring areas pose unusual health challenges when an epidemic originates from this region. While normally in South Korea, the spread of diseases such as ASF and HPAI will be monitored, modeled, predicted, and controlled [13, 63], there is a general dearth of data in the DMZ. As a result in this area, it is impossible to monitor and gather pertinent information on wild animals' movement and the spread of viruses inside. Furthermore, due to national security reasons, large-scale development in the neighboring areas is prohibited; as a result, livestock farming has become a major industry in the region.

**Figure 4:**
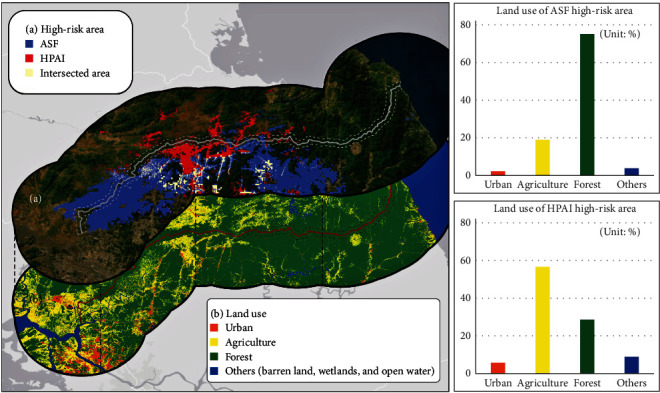
Distribution of land use and high-risk areas of ASF and HPAI.

Wild boars are primary hosts of ASF, and these are typically found in forests where the risk of ASF outbreaks is the highest [64, 65, 66]. Forests provide an ideal environment for wild boars to survive, implying that forests, especially in the northern region, facilitate ASF spreads in South Korea [22, 67]. Furthermore, the peninsula's dry and cold winter encouraged wild boars to mobilize even more so that they could maintain their body temperature and secure food [63]. However, our results showed that land use did not significantly contribute to explaining the ASF spreads; instead, precipitation, mean temperature, and solar radiation played significant roles (Tables 2 and S3), although the majority of ASF-positive cases were found in forest areas (Figures 4 and S3; Table 3). In other words, drivers of ASF spread and known habitat preferences of wild boars might not necessarily agree; hence, understanding only their habitat preferences may not be sufficient in preventing ASF epidemics.

Previous research has shown a positive relationship between HPAI with agricultural land that provides seasonal habitat and food availability [29, 68, 69]. The habitats and migratory patterns of birds are significantly affected by temperature sensitivity [70]. Particularly during winter, migratory birds flock to agricultural areas with abundant food sources, which further increases the risk of HPAI outbreaks. Our MaxEnt results also support the relationship between HPAI with agricultural land (Table 2; Figure S4). We observed an important lag effect as December precipitation significantly influenced the timing of HPAI outbreaks some 1–3 months later. In our PCA, December's precipitation of PC2 showed a higher loading, similar to the loadings of January, February, and March (Table S4). April did not show any similar influence. This is consistent with other research identifying a time lag between rainfall and wild bird-mediated avian influenza virus [71, 72].

Both ASF and HPAI high-risk areas were closely located to the livestock industry (pork and poultry farms) of South and North Korea. Most of the ASF high-risk areas in the South were distributed in Paju, Yeoncheon, and Hwacheon (Figure S5). These regions were characterized by the concentration of pork farms in each province. Cheolwon, where HPAI high-risk areas of South Korea were concentrated (Figure S5), produced the highest number of chickens in Gangwon province [73]. On the other hand, ASF and HPAI high-risk areas in the North included the largest livestock farm in the Sepho district (Figure S6). Based on our risk maps (Figures 3 and 4), large areas of the DMZ are susceptible to ASF and HPAI transmission. This finding is consistent with previous findings highlighting the DMZ as a wildland habitat that also contained reservoirs for ASF and HPAI [12]. Moreover, HPAI consistently cross species barriers, posing a threat to both birds and mammals [74]. The diverse ecosystem within DMZ creates conditions conducive to the emergence of novel strains of HPAI.

ASF being endemic in South Korea inherently means that it is also endemic in our study area, i.e., the inner-border region. In contrast, HPAI is not endemic in South Korea, so it is difficult to uphold the equilibrium assumption when MaxEnt was to be applied to the entire South Korea. HPAI may not be fully endemic in our study area, but it still may be portrayed as a series of localized outbreaks, i.e., “semi-endemic.” However, our situation might be even more limited because of data inaccessibility; it is probable that some might regard our HPAI data as not being large enough to fully depict this semi-endemic situation. Yet, we have applied MaxEnt to our limited HPAI data because it is the best spatially explicit data available. Similarly, a recent Chinese case study employed only 200 data points to cover the entire mainland China (9,597,000 km^2^ in area), which means that each data point covers 47,985 km^2^ to analyze HPAI risks between 2014 and 2021 [75]. This provides a precedent that MaxEnt is capable of yielding reliable and robust results even with limited data. In our case study, each data point covers a far finer resolution of 103.61 km^2^ (144 data points, 14,921 km^2^) and only for 2 months.

While we were not able to employ virus data from North Korea, our MaxEnt results provide estimates of the potential risk based on the common environmental characteristics contributing to disease spread [76] by extrapolating risks in the South to the North. As our research was not able to use any ASF data points from North Korea, it was reasonable to believe that the lower risk in North Korea could be an artifact. Nevertheless, the western region in North Korea showed many high-risk areas, implying that there are suitable ecological niches for ASF to spread, and as such makes it an area of concern (Figure 3(a)). The DMZ was also an area of concern because it included more areas with high (16.84%) and moderate (34.07%) risks than North Korea (Table 4). This result might be partly due to HPAI having fewer cases than ASF and also due to the possibility that the study area was less suitable for HPAI to spread than ASF. Identical to the ASF case, our research was not able to use any HPAI data points from North Korea, and yet more high-risk areas were identified in North Korea for the HPAI, as compared to ASF. Our MaxEnt models presented high AUC values for both training and test data, suggesting more reliable risk predictions [77] and further solidifying this type of modeling approach in low-data environments [78, 79]. However, the accuracy of the MaxEnt model might only work for the South, as the model's predictive accuracy could be lower in North Korea.

To our knowledge, our research was the first to study the simultaneous risk of ASF and HPAI. These two highly contagious animal diseases were not often studied in tandem because they were unrelated in terms of viral etiology [80, 81]; also, our data showed these epidemics occurred in different time periods. In the DMZ, adopting an all-hazard threat approach, local municipalities and residents still need to be prepared for the simultaneous outbreak of these viruses. Moreover, integrating the DMZ infectious disease risk map with data on the movement of individuals and vehicles in the area can furnish essential insights into how the infectious disease risk within the DMZ might influence the future spread of diseases within the country.

## 5. Conclusions

Our research furthers understanding of the geographic patterns of ASF and HPAI in the DMZ region, and the environmental factors contributing to their distribution. Our study found that the MaxEnt model accurately predicted the occurrence of both diseases, even with the limited occurrence data. Despite the current lower risk of ASF in North Korea compared to South Korea, both countries face significant threats from these diseases.

Our MaxEnt variable contribution analysis highlighted a correlation between virus distributions and the habitats of disease vectors. More precipitation and sunlight and higher temperature were the primary factors driving ASF spread, while precipitation, temperature, and land use were critical for the HPAI epidemic. Drivers of ASF spread and known habitat preferences of wild boars may not necessarily agree because our MaxEnt outcome showed that land use contributed little in explaining the ASF spread.

Given the DMZ's unique context, further research is needed to assess the impact of wildlife populations and border restrictions on transboundary disease transmission. Such research would aid in developing targeted prevention strategies. Overall, our study emphasizes the importance of considering environmental factors and land use patterns when assessing disease spread risks in border regions. While developing disease surveillance strategies between South and North Korea that would more accurately capture the level of any future outbreak, the reality is that this will not happen. Results such as those presented here are vital to help formulate effective measures to mitigate the impact of these diseases on animal and human health.

## Figures and Tables

**Table 1:** Environmental variables.

Environmental variable	Resolution	Unit	Source
Mean temperature (monthly)	1 km	°C	WorldClim, https://www.worldclim.org
Precipitation (monthly)	1 km	mm	
Solar radiation (monthly)	1 km	$\frac{\text{kJ}}{m^{2}\cdot \text{day }}$	

Elevation	30 m	m	Ministry of Environment, https://egis.me.go.kr
Land use (categorical)	30 m	—	

**Table 2:** MaxEnt results.

		ASF		HPAI	
		Initial	Final	Initial	Final
Permutation importance (%)	PC1	23.05	22.87	45.83	37.88
	PC2	17.88	18.26	43.02	48.09
	PC3	30.84	31.97	2.44	—
	PC4	26.36	26.90	—	—
	Land use	1.88	—	8.72	14.02

AUC	Training	0.918	0.915	0.988	0.989
	Testing	0.920	0.917	0.989	0.987

**Table 3:** Area and ratio of land uses regarding high- and low-risk areas of ASF and HPAI.

Land type	ASF				HPAI			
	High-risk		Low-risk		High-risk		Low-risk	
	Area (km^2^)	Ratio (%)	Area (km^2^)	Ratio (%)	Area (km^2^)	Ratio (%)	Area (km^2^)	Ratio (%)
Urban	45.23	2.20	286.96	3.25	35.93	5.75	333.34	2.83
Agriculture	388.28	18.92	2,058.79	23.33	354.11	56.64	2,258.80	19.21
Forest	1,539.94	75.05	6,081.57	68.93	179.24	28.67	8,717.49	74.14
Others	78.54	3.83	396.06	4.46	55.92	8.94	448.73	3.82
Total	2,051.98	100.00	8,823.38	100.00	625.20	100.00	11,758.36	100.00

**Table 4:** Area and ratio of risks for ASF and HPAI.

		South Korea		North Korea		DMZ	
		Area (km^2^)	Ratio (%)	Area (km^2^)	Ratio (%)	Area (km^2^)	Ratio (%)
ASF	High	1,657.36	26.88	244.33	3.98	151.15	16.84
	Moderate	1,518.04	24.62	500.39	8.15	305.75	34.07
	Low	2,990.59	48.50	5,391.44	87.86	440.52	16.84

HPAI	High	432.37	7.01	142.25	2.32	51.01	5.68
	Moderate	459.13	7.45	268.85	4.38	88.58	9.87
	Low	5,274.48	85.54	5,725.06	93.30	757.83	84.45

## Data Availability

Access to data that are used in this research is restricted. One may have to contact the central and local governments to access the data.

## Supplementary Materials

Supplementary Materials Additional information of PCA and MaxEnt analyses.
