# Supplementary material for: Mapping African Swine Fever and Highly Pathogenic Avian Influenza Outbreaks along the Demilitarized Zone in the Korean Peninsula
Source: Transbound Emerg Dis. 2024 May 30;2024:8824971. doi: 10.1155/2024/8824971 (PMC12017379; doi:10.1155/2024/8824971)
Supplement: Supplementary Materials — Additional information of PCA and MaxEnt analyses. [file 8824971.f1.docx]

Table S1. PCA results for ASF (selected PCs highlighted in grey).

| PC | Total | Variance (%) | Cumulative variance (%) |
| --- | --- | --- | --- |
| 1 | 20.82 | 56.27 | 56.27 |
| 2 | 8.81 | 23.81 | 80.08 |
| 3 | 3.55 | 9.61 | 89.63 |
| 4 | 1.35 | 3.66 | 93.35 |
| 5 | 1.14 | 3.08 | 96.42 |
| 6 | 0.43 | 1.18 | 97.60 |

Table S2. PCA results for HPAI (selected PCs highlighted in grey).

| PC | Total | Variance (%) | Cumulative variance (%) |
| --- | --- | --- | --- |
| 1 | 8.98 | 56.15 | 56.15 |
| 2 | 3.90 | 24.36 | 80.50 |
| 3 | 1.74 | 10.87 | 91.38 |
| 4 | 0.70 | 4.38 | 95.76 |
| 5 | 0.39 | 2.42 | 98.18 |
| 6 | 0.09 | 0.58 | 98.76 |

Table S3. Environmental variables and their factor loadings for ASF (higher loading values highlighted in grey).

| **Predictor variable** | **Component** | | | |  |
| --- | --- | --- | --- | --- | --- |
|  | **1** | **2** | **3** | **4** | **5** |
| tavg_01 | 0.155 | 0.229 | 0.009 | 0.049 | 0.090 |
| tavg_02 | 0.183 | 0.178 | -0.009 | 0.101 | 0.066 |
| tavg_03 | 0.198 | 0.126 | -0.039 | 0.157 | 0.031 |
| tavg_04 | 0.202 | 0.093 | -0.050 | 0.208 | 0.040 |
| tavg_05 | 0.205 | 0.059 | -0.060 | 0.227 | -0.003 |
| tavg_06 | 0.211 | -0.004 | -0.024 | 0.220 | -0.011 |
| tavg_07 | 0.211 | 0.016 | -0.021 | 0.215 | 0.010 |
| tavg_08 | 0.213 | 0.032 | -0.009 | 0.186 | -0.013 |
| tavg_09 | 0.210 | 0.071 | -0.017 | 0.149 | -0.024 |
| tavg_10 | 0.192 | 0.154 | -0.033 | 0.116 | 0.000 |
| tavg_11 | 0.168 | 0.211 | -0.019 | 0.092 | 0.055 |
| tavg_12 | 0.150 | 0.238 | -0.013 | 0.058 | 0.093 |
| srad_01 | 0.143 | 0.097 | 0.152 | -0.360 | 0.430 |
| srad_02 | 0.189 | -0.053 | 0.044 | -0.274 | 0.240 |
| srad_03 | 0.197 | -0.093 | -0.018 | -0.244 | -0.121 |
| srad_04 | 0.198 | -0.055 | 0.147 | -0.140 | -0.189 |
| srad_05 | 0.195 | -0.114 | 0.045 | -0.125 | -0.233 |
| srad_06 | 0.190 | -0.135 | 0.031 | -0.117 | -0.222 |
| srad_07 | 0.185 | 0.037 | 0.093 | -0.239 | -0.265 |
| srad_08 | 0.199 | -0.083 | 0.093 | -0.105 | -0.220 |
| srad_09 | 0.194 | -0.120 | 0.044 | -0.096 | -0.235 |
| srad_10 | 0.201 | -0.083 | 0.115 | -0.101 | -0.144 |
| srad_11 | 0.193 | 0.039 | 0.159 | -0.191 | 0.090 |
| srad_12 | 0.157 | 0.126 | 0.157 | -0.235 | 0.361 |
| prec_01 | -0.092 | 0.260 | 0.131 | 0.032 | -0.335 |
| prec_02 | -0.122 | 0.246 | 0.194 | 0.016 | -0.063 |
| prec_03 | -0.071 | 0.113 | 0.450 | 0.101 | -0.098 |
| prec_04 | -0.082 | 0.011 | 0.477 | 0.058 | 0.039 |
| prec_05 | 0.016 | -0.148 | 0.439 | 0.207 | 0.127 |
| prec_06 | -0.100 | -0.166 | 0.326 | 0.188 | 0.042 |
| prec_07 | 0.021 | -0.315 | -0.002 | 0.092 | 0.006 |
| prec_08 | 0.100 | -0.202 | 0.225 | 0.223 | 0.025 |
| prec_09 | -0.095 | 0.289 | 0.037 | -0.025 | 0.054 |
| prec_10 | -0.073 | 0.290 | 0.068 | -0.104 | -0.151 |
| prec_11 | -0.028 | 0.311 | 0.093 | -0.046 | -0.179 |
| prec_12 | -0.144 | 0.235 | -0.019 | -0.093 | -0.197 |
| elev | -0.204 | -0.064 | 0.088 | -0.159 | 0.008 |

Table S4. Environmental variables and their factor loadings for HPAI (higher loading values highlighted in grey).

| **Predictor**  **variable** | **Component** | | |
| --- | --- | --- | --- |
|  | **1** | **2** | **3** |
| tavg_01 | 0.265 | 0.284 | 0.143 |
| tavg_02 | 0.297 | 0.205 | 0.131 |
| tavg_03 | 0.311 | 0.122 | 0.140 |
| tavg_04 | 0.312 | 0.074 | 0.135 |
| tavg_12 | 0.258 | 0.290 | 0.180 |
| srad_01 | 0.248 | 0.139 | -0.294 |
| srad_02 | 0.291 | -0.111 | -0.244 |
| srad_03 | 0.282 | -0.181 | -0.132 |
| srad_04 | 0.277 | -0.062 | -0.273 |
| srad_12 | 0.266 | 0.186 | -0.237 |
| prec_01 | -0.125 | 0.434 | 0.126 |
| prec_02 | -0.161 | 0.438 | -0.003 |
| prec_03 | -0.114 | 0.352 | -0.420 |
| prec_04 | -0.138 | 0.220 | -0.564 |
| prec_12 | -0.191 | 0.343 | 0.252 |
| elev | -0.313 | -0.018 | -0.156 |

Figure S1. Scree plot of environmental variable factor loading for ASF.

Figure S2. Scree plot of environmental variable factor loading for HPAI.


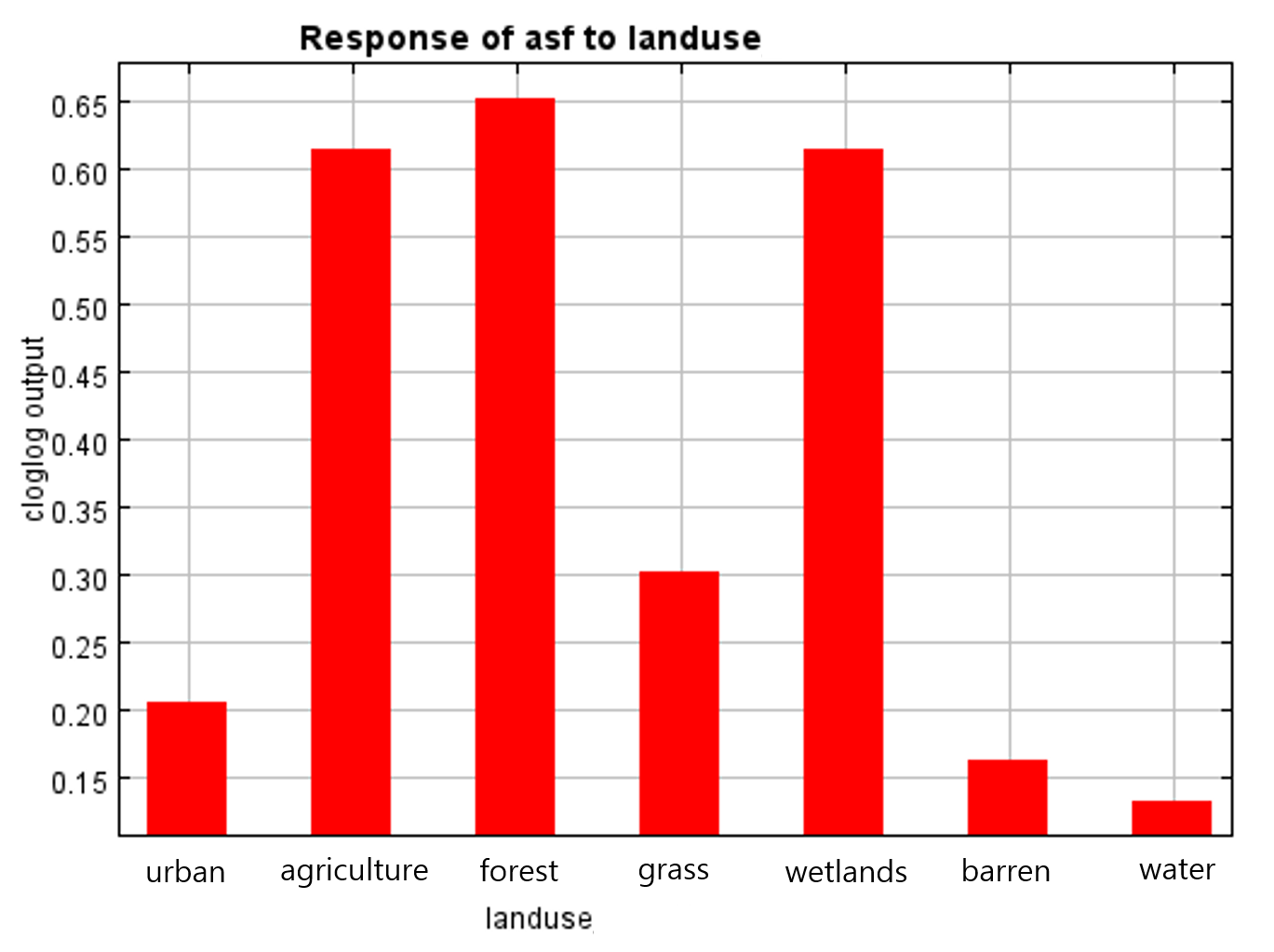


Figure S3. ASF MaxEnt's response histogram for land uses.


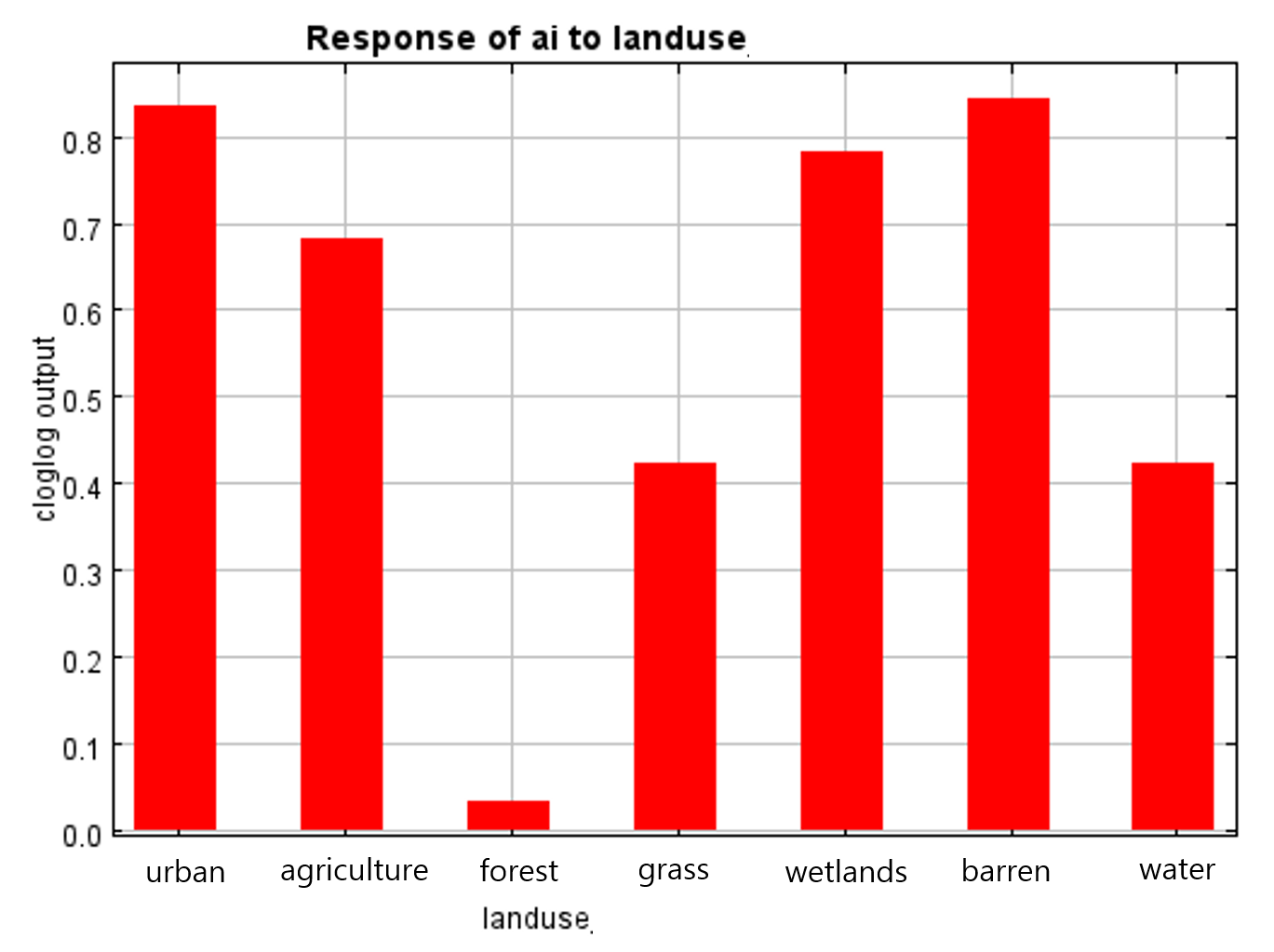


Figure S4. HPAI MaxEnt's response histogram for land uses.


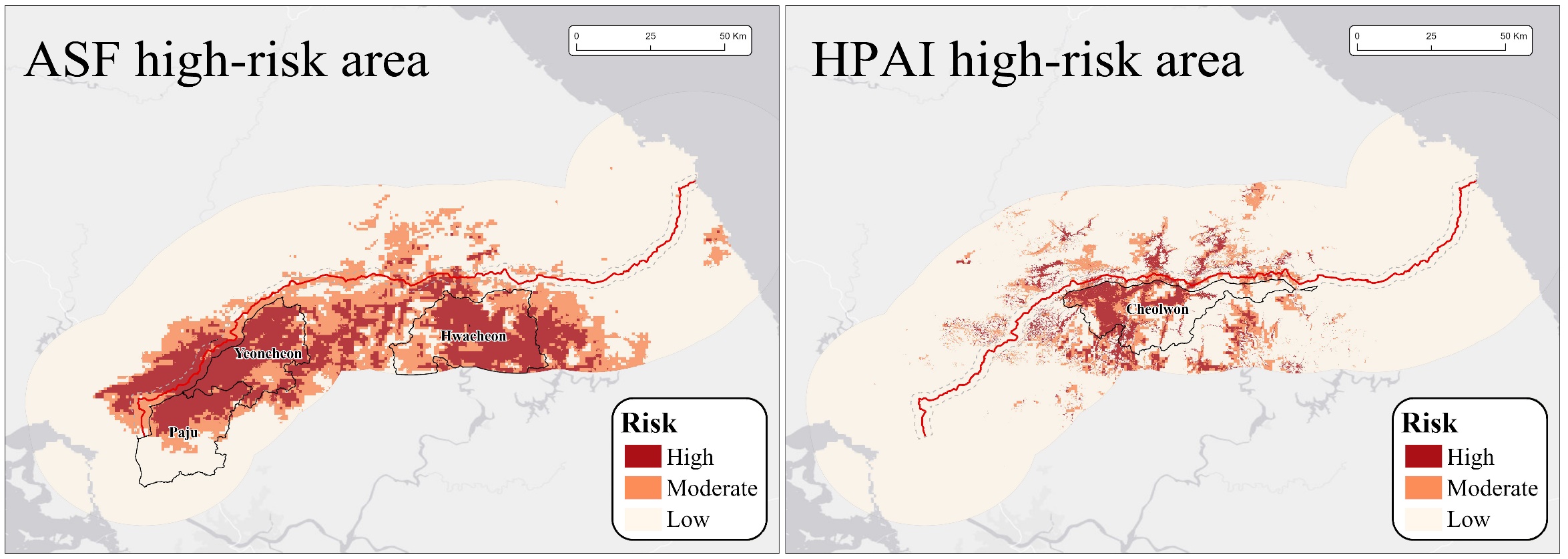


Figure S5. ASF and HPAI high-risk areas by municipalities in South Korea.


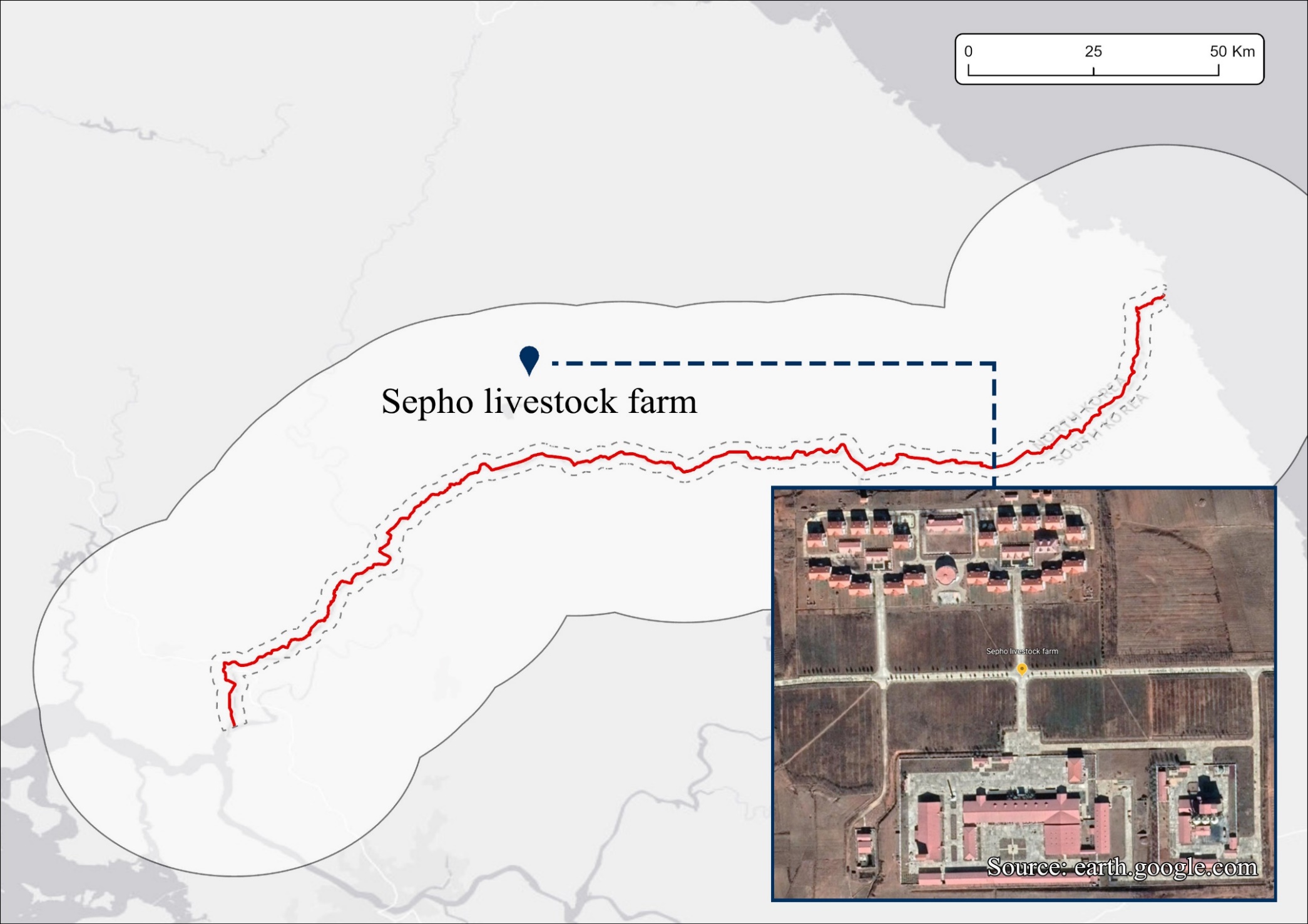


Figure S6. Location of Sepho livestock farm in North Korea (Latitude: 38.4704374N, Longitude: 127.2190176E in decimal degrees).
